# Standing geographic variation in eclosion time and the genomics of host race formation in *Rhagoletis pomonella *fruit flies

**DOI:** 10.1002/ece3.4758

**Published:** 2018-12-14

**Authors:** Meredith M. Doellman, Scott P. Egan, Gregory J. Ragland, Peter J. Meyers, Glen R. Hood, Thomas H. Q. Powell, Peter Lazorchak, Daniel A. Hahn, Stewart H. Berlocher, Patrik Nosil, Jeffrey L. Feder

**Affiliations:** ^1^ Department of Biological Sciences University of Notre Dame Notre Dame Indiana; ^2^ Advanced Diagnostics and Therapeutics Initiative University of Notre Dame Notre Dame Indiana; ^3^ Department of Biosciences Rice University Houston Texas; ^4^ Environmental Change Initiative University of Notre Dame Notre Dame Indiana; ^5^ Department of Integrative Biology University of Colorado–Denver Denver Colorado; ^6^ Department of Biological Sciences Wayne State University Detroit Michigan; ^7^ Department of Biological Sciences State University of New York–Binghamton Binghamton New York; ^8^ Department of Computer Science Johns Hopkins University Baltimore Maryland; ^9^ Department of Entomology and Nematology University of Florida Gainesville Florida; ^10^ Department of Entomology University of Illinois at Urbana‐Champaign Urbana Illinois; ^11^ Department of Animal and Plant Sciences University of Sheffield Sheffield UK

**Keywords:** clinal variation, eclosion time, ecological speciation, host races, standing variation

## Abstract

Taxa harboring high levels of standing variation may be more likely to adapt to rapid environmental shifts and experience ecological speciation. Here, we characterize geographic and host‐related differentiation for 10,241 single nucleotide polymorphisms in *Rhagoletis pomonella *fruit flies to infer whether standing genetic variation in adult eclosion time in the ancestral hawthorn (*Crataegus* spp.)‐infesting host race, as opposed to new mutations, contributed substantially to its recent shift to earlier fruiting apple (*Malus domestica*). Allele frequency differences associated with early vs. late eclosion time within each host race were significantly related to geographic genetic variation and host race differentiation across four sites, arrayed from north to south along a 430‐km transect, where the host races co‐occur in sympatry in the Midwest United States. Host fruiting phenology is clinal, with both apple and hawthorn trees fruiting earlier in the North and later in the South. Thus, we expected alleles associated with earlier eclosion to be at higher frequencies in northern populations. This pattern was observed in the hawthorn race across all four populations; however, allele frequency patterns in the apple race were more complex. Despite the generally earlier eclosion timing of apple flies and corresponding apple fruiting phenology, alleles on chromosomes 2 and 3 associated with earlier emergence were paradoxically at lower frequency in the apple than hawthorn host race across all four sympatric sites. However, loci on chromosome 1 did show higher frequencies of early eclosion‐associated alleles in the apple than hawthorn host race at the two southern sites, potentially accounting for their earlier eclosion phenotype. Thus, although extensive clinal genetic variation in the ancestral hawthorn race exists and contributed to the host shift to apple, further study is needed to resolve details of how this standing variation was selected to generate earlier eclosing apple fly populations in the North.

## 1 INTRODUCTION

The raw material for novel adaptation can come from new mutations or standing variation (Barrett & Schluter, 2008). Adaptation is expected to occur more rapidly when based on standing variation, because the process is not limited by wait time for new beneficial mutations to arise and standing variants are likely to have been filtered for negative pleiotropic fitness effects or detrimental epistatic interactions (Barrett & Schluter, 2008; Yeaman, 2015). Therefore, taxa harboring high levels of standing genetic variation may be more likely to adapt to rapid environmental shifts and experience ecological speciation than those requiring new favorable mutations to respond. Indeed, myriad examples are accumulating of rapid adaptive evolution fueled by standing variation, including coat color in the old field mouse, *Peromyscus polionotus* (Steiner, Weber, & Hoekstra, 2007), reduced defensive armor in the threespine stickleback fish, *Gasterosteus aculeatus* (Colosimo et al., 2005), mimicry in *Heliconius* butterflies (The Heliconius Genome Consortium, 2012), and beak morphology in Darwin’s Finches (Lamichhaney et al., 2015). Standing variation may be especially relevant in cases where adaptation involves polygenic traits, and the majority of favorable alleles have only minor effects on fitness and genomic incompatibility (Barrett & Schluter, 2008; Hermisson & Pennings, 2005; Schluter & Conte, 2009). Large stores of standing genetic variation observed in some populations may be a product of complex evolutionary histories, including past gene flow coupled with ecological selection to maintain high levels of genetic variation resulting from tracking local, regional, or temporal differences in environmental or biotic conditions (Bergland, Tobler, González, Schmidt, & Petrov, 2016; Berner & Salzburger, 2015; Brawand et al., 2014; Feder, Berlocher, et al., 2003; Gompert, Fordyce, Forister, Shapiro, & Nice, 2006; Jeffery et al., 2017; Jones et al., 2012; Lamichhaney et al., 2015; Loh et al., 2013; Pease, Haak, Hahn, & Moyle, 2016; Roesti, Gavrilets, Hendry, Salzburger, & Berner, 2014; The Heliconius Genome Consortium, 2012).

Reservoirs of standing genetic variation are often maintained across geographic clines, gradients of phenotypic or genetic change in populations across space that can provide windows into understanding adaptive evolution and speciation (Endler, 1977; Huxley, 1938). Geographic clines can be either primary, due to selection across a landscape, or secondary, as a result of population subdivision followed by hybridization and introgression of loci. In either case, analysis of the differential spatial distribution of phenotypes and loci can help identify traits and genes involved in adaptation that may contribute to reproductive isolation (Barton & Gale, 1993; Barton & Hewitt, 1981, 1985; Gompert, Mandeville, & Buerkle, 2017; Harrison & Larson, 2016; Jiggins & Mallet, 2000; Kruuk, Baird, Gale, & Barton, 1999; Mallet et al., 1990; Payseur, 2010; Slatkin, 1973; Szymura & Barton, 1986, 1991). However, clines can be indicative of more than just past progress toward speciation but also be natural experiments in the speciation process itself, with gene flow providing an input of new material facilitating divergence (Abbott et al., 2013; Arnold, 1997; Endler, 1977; Gompert et al., 2014; Harrison & Larson, 2016; Hewitt, 1988; Mallet, 2007). In this regard, hybridization can create novel genotypes that may rapidly lead to differentiation from nearby parental taxa when hybrids occupy underused niches or environments, via polyploid or homoploid mechanisms (Gompert et al., 2006; Kang, Schartl, Walter, & Meyer, 2013; Lamichhaney et al., 2018; Mavárez et al., 2006; Rieseberg & Willis, 2007; Salzburger, Baric, & Sturmbauer, 2002; Schwarz, Matta, Shakir‐Botteri, & McPheron, 2005; Yakimowski & Rieseberg, 2014). In addition, hybridization need not have an immediate effect on generating new taxa (Abbott, Barton, & Good, 2016) but can also create and maintain extensive standing variation, enabling divergence at a later time when populations experience new ecological opportunities (Berner & Salzburger, 2015).

*Rhagoletis pomonella* (Diptera: Tephritidae), a model for population divergence in sympatry, provides an avenue for investigating the role of standing clinal variation in rapid adaptation and speciation in response to novel ecological opportunity (Berlocher & Feder, 2002). Ancestral *R. pomonella* infested the fruits of native North American hawthorns (*Crataegus* spp.) and shifted (<170 years ago) to domesticated apple (*Malus domestica*) after the plant was introduced by European settlers ~400 years ago (Bush, 1966; Walsh, 1867). Previous studies have implied that hawthorn‐infesting populations of *R. pomonella* possess large stores of phenotypic and genetic variation, notably for life history timing, which may have facilitated the host shift to apple (Feder et al., 2005; Feder, Berlocher, et al., 2003). In particular, differences in the timing of adult eclosion have been shown to be an important host‐related ecological adaptation contributing to partial allochronic premating isolation between apple and hawthorn flies (Feder et al., 1994; Feder, Hunt, & Bush, 1993). Fruit on apple varieties favorable for larval survivorship ripens about 3–4 weeks earlier than those of downy hawthorn (*C. mollis*), the primary host of *R. pomonella* in the Midwestern and Northeastern United States (Feder et al., 1994). Flies must synchronize breakage of pupal diapause and adult eclosion with the availability of ripe host fruit for mating and oviposition. This is critical because *Rhagoletis* is univoltine, adults take a week to reach sexual maturity, and flies live for a maximum of 1 month in nature (Dean & Chapman, 1973). As a result, apple flies have evolved to eclose an average of 10 days earlier than hawthorn flies, as measured in field capture studies, reducing host race overlap to ~80% (Feder et al., 1993, 1994). This partial allochronic isolation, in combination with host fidelity (e.g., host fruit odor preference), reduces gene flow between the host races to ~4%–6% per generation (Feder et al., 1993, 1994).

**Figure 1:**
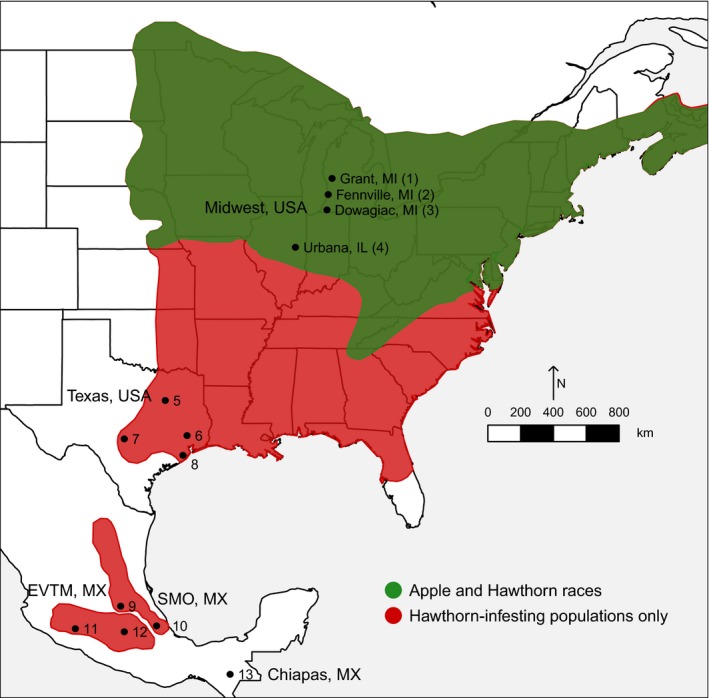
Map of the four paired sympatric collection sites for apple and hawthorn flies in the Midwestern United States. Also given are the ranges of the apple race and native hawthorn‐infesting populations of *Rhagoletis pomonella* in the United States and Mexico (see Supporting Information Table S2 for numerical designations of populations and site information). Note that the 430‐km transect through the Midwestern United States encompasses much of the latitudinal range of overlap of the apple and hawthorn host races in the region. The primary hawthorn host of *R. pomonella* in the Northeastern and Midwestern United States is *Crataegus mollis*. However, moving south from Urbana, apple is not infested and *C. mollis* becomes rare, although a variety of the species, *C. mollis texana*, exists in the state of Texas, United States (site 5). Other hawthorn species, with varying fruiting times, are the primary hosts of the fly in the southern United States and Mexico (see Figure 2a and Supporting Information Table S2; Lyons‐Sobaski & Berlocher, 2009; Rull et al., 2006). DNA sequencing data imply that hawthorn‐infesting *R. pomonella* populations from the Eje Volcánico Trans Mexicano (EVTM) and those in the Sierra Madre Oriental Mountains of Mexico (SMO) and United States have undergone cycles of allopatry followed by secondary contact and gene flow over the past ~1.5 My (Feder et al., 2005; Feder, Berlocher, et al., 2003; Michel et al., 2007; Xie et al., 2007), contributing to the creation and maintenance of geographic genetic variation in eclosion time in the fly

**Figure 2:**
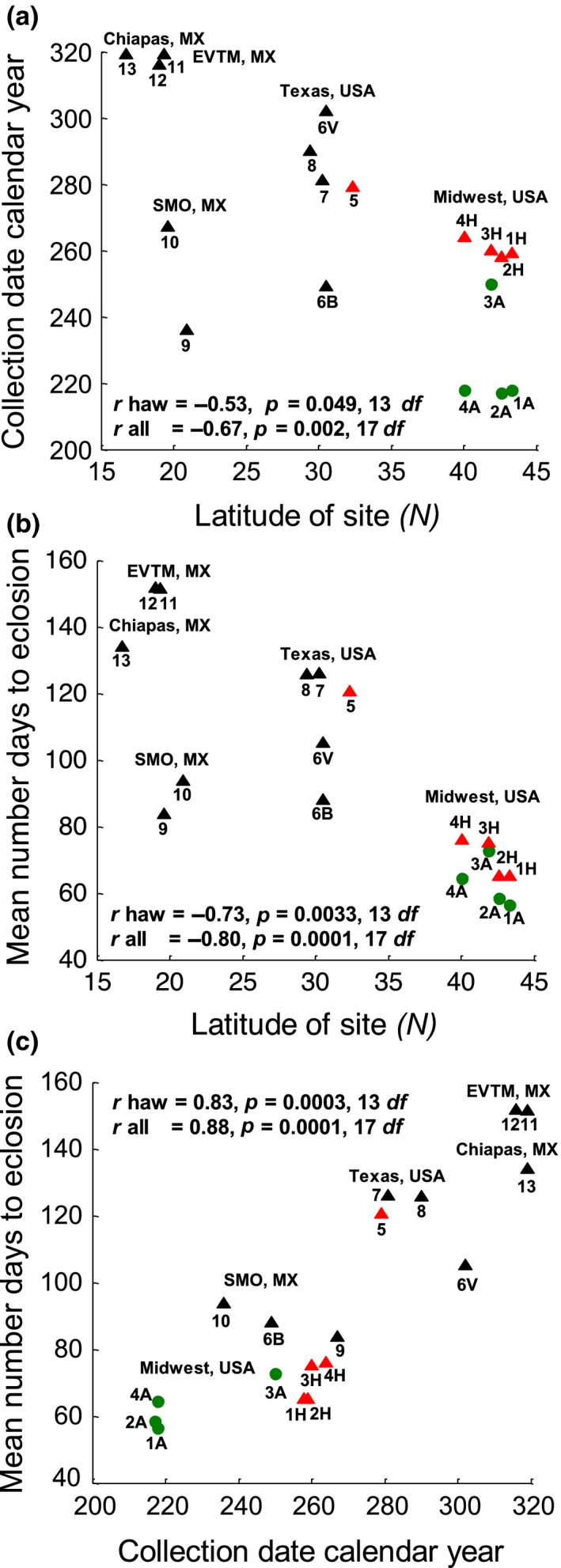
Geographic variation in host fruiting phenology and fly eclosion time along a latitudinal transect of 18 different *Rhagoletis pomonella* populations analyzed at 13 sites from Grant, MI, United States, to the highlands of Chiapas, Mexico (MX) (Data are from Dambroski & Feder, 2007; Hood et al., 2015; Lyons‐Sobaski & Berlocher, 2009; Xie et al., 2007; see Figure 1 for a map of the 13 sampled sites and Supporting Information Table S2 for population designations and additional information. Sites with the same number but different letters represent flies sampled from different hosts at the site). (a) Host fruiting time, as indicated by the collecting date of fly populations at sites, plotted against latitude; (b) mean time to eclosion measured as the average number of days following postwinter warming of fly populations determined from controlled laboratory rearing studies plotted against latitude; and (c) mean eclosion time of fly populations plotted against host fruiting time at sites. Grant, MI (site 1), and Urbana, IL (site 4), represent the approximate northernmost and southernmost ends, respectively, where apple (green circles) and *Crataegus mollis* (red triangles) host races geographically overlap in the Midwestern United States. Note that flies from site 5 infest *C. mollis v. texana* in Texas which is also depicted with a red triangle, while other hawthorn‐infesting fly populations are shown in black triangles. Geographic and host‐related differentiation in host fruiting time and fly eclosion in the Midwestern United States is subsumed within a larger pattern of variation across North America. There is a general trend for both host fruit to ripen and flies to eclose later in the year with decreasing latitude, as evidenced by significant negative correlations (*r* values are given considering the hawthorn data alone = *r*, and for all hosts including apples = *r* all). The relationship is complicated, however, by *R. pomonella* attacking different hawthorn species moving southward from the Midwest (see Supporting Information Table S2) that fruit at varying times during the season (Lyons‐Sobaski & Berlocher, 2009; Rull et al., 2006; Xie et al., 2007), as well as altitudinal effects in Mexico

**Figure 3:**
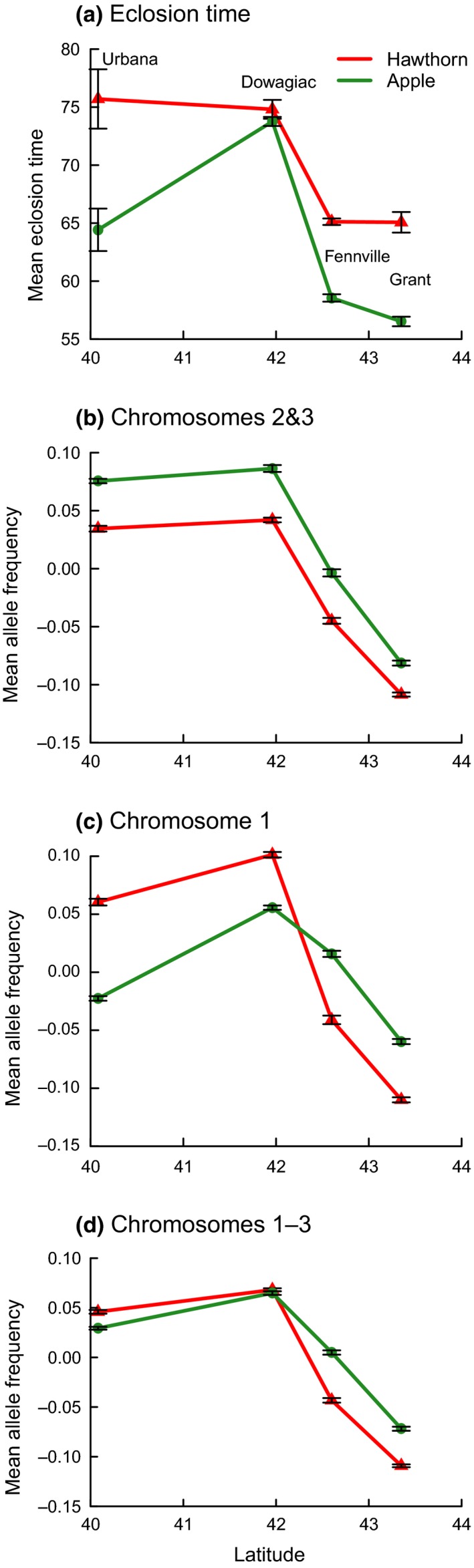
(a) Mean time to eclosion measured as the average number of days following postwinter warming for apple and hawthorn flies in controlled rearing experiments (data from Hood et al., 2015; see also Supporting Information Table S2 for additional site information) and b–d) mean standardized allele frequencies for SNP variants significantly associated with later eclosion time in the GWAS of Ragland et al. (2017) at the four latitudinally arrayed study sites surveyed across the Midwestern United States. Populations having higher mean standardized allele frequencies are therefore predicted to be genetically inclined to eclose later than populations having lower mean standardized allele frequencies. Panel (b) chromosome 2 and 3 SNPs together; (c) chromosome 1 SNPs; (d) chromosome 1–3 SNPs together; see Materials and Methods for details concerning the calculation of mean standardized allele frequencies for SNPs. *n* = number of significant SNPs on chromosome(s) contributing to mean standardized allele frequencies

Eclosion time appears to have a complex evolutionary history associated with standing clinal variation in the ancestral hawthorn race (Feder & Bush, 1989; Feder, Berlocher, et al., 2003; Feder, Chilcote, & Bush, 1990; Michel et al., 2010; Michel, Rull, Aluja, & Feder, 2007). It has been hypothesized that part of the variation originated in the Eje Volcánico Trans Mexicano (EVTM) ~1.5 million years ago, during a period of isolation from other Mexican and North American populations (see Figure 1; Michel et al., 2007; Xie et al., 2007). It then spread through the Sierra Madre Oriental Mountains (SMO) of Mexico and into North America over the past ~1 million years via episodes of secondary contact and introgression between hawthorn fly populations in Mexico and the United States (Feder et al., 2005; Feder, Berlocher, et al., 2003; Michel et al., 2007; Xie et al., 2007). Subsequently, selection related to latitudinal, altitudinal, and species differences in hawthorn fruiting time in the SMO and United States likely maintained this adaptive variation in eclosion timing*. *Consistent with this scenario, many species of hawthorns ripen later in the year with decreasing latitude, reflected in later dates of fly collection (Figure 2a; Lyons‐Sobaski & Berlocher, 2009; Rull, Aluja, Feder, & Berlocher, 2006; Xie et al., 2007). As a result, eclosion time varies geographically, with hawthorn flies from further south requiring more time to eclose than those from further north, both in nature and in controlled rearing experiments (Figures 2b and 3a; Dambroski & Feder, 2007; Hood et al., 2015; Lyons‐Sobaski & Berlocher, 2009; Xie et al., 2007). This standing geographic variation in eclosion timing of hawthorn flies is thought to have contributed to the adaptive radiation of the *R. pomonella* sibling species group by allowing these short‐lived, univoltine flies to attack novel host plants with differing fruiting times, including the formation of a number of races and potentially species on different hawthorns in southern latitudes (Powell, Cha, Linn, & Feder, 2012; Powell, Forbes, Hood, & Feder, 2014) and most recently the apple race in the Eastern United States (Feder, Chilcote, & Bush, 1988).

Previous studies from our group provided evidence for a link between standing clinal variation and host race formation but had insufficient genomic resolution to infer the genetic architecture of either the underlying eclosion time phenotypes or host race differentiation (Feder & Bush, 1989; Feder et al., 1990, 2005; Feder, Berlocher, et al., 2003; Michel et al., 2007, 2010; Xie et al., 2008, 2007). Ragland et al. (2017) recently laid a genomic foundation for understanding the architecture of eclosion timing and its association with host race differentiation in *R. pomonella* (see Supporting Information Appendix S1 for additional details). In a genome‐wide association study (GWAS) of adult eclosion time in hawthorn and apple flies collected from a field site in Fennville, Michigan (MI), United States, Ragland et al. (2017) compared single nucleotide polymorphism (SNP) allele frequency differences between the earliest (≤3%) and latest (≥97%) eclosing quantiles of flies. They found that SNPs displaying significant allele frequency differences between early‐ and late‐eclosing flies in both host races were concentrated on three of the five major chromosomes (numbers 1–3) constituting the *R. pomonella* genome (Supporting Information Table S1). Within chromosomes 1–3, SNPs displaying high levels of linkage disequilibrium (LD) with one another, presumably due to chromosomal inversions (Feder, Roethele, Filchak, Niedbalski, & Romero‐Severson, 2003b), showed the greatest frequency responses (Table S1; see Supporting Information Appendix S1 for further discussion of inversions and their origins). However, a proportion of low LD SNPs on chromosomes 1–3 also displayed significant responses above random expectation in the GWAS (Supporting Information Table S1). These low LD SNPs presumably represent loci in more freely recombining, colinear regions of chromosomes also contributing to eclosion timing.

The highly polygenic nature of eclosion time demonstrated by Ragland et al. (2017) provides additional evidence supporting the hypothesis that standing genetic variation, rather than new mutations, likely fueled *R. pomonella’*s shift to the earlier fruiting apple host. However, a stronger case would be made if allele frequencies for SNPs responding in the eclosion time GWAS also showed significant and predictable geographic and host‐related variation in nature. Here, we investigate genome‐wide patterns of differentiation in *Rhagoletis pomonella *(Diptera: Tephritidae) for 10,241 SNPs by comparing results from the adult eclosion GWAS (Ragland et al., 2017) with a population survey of four sites distributed from north to south along a 430‐km transect across the Midwestern United States, where populations of hawthorn and apple flies co‐occur in sympatry (Figure 1). We determine the degree to which the responses of eclosion‐associated SNPs from the GWAS predict geographic allele frequency differences within the host races across the Midwest and local, host‐related differences between apple and hawthorn fly populations at the four sympatric sites.

In this study, we test four specific predictions of the standing variation hypothesis, regarding how genetic variation for eclosion time should be associated with geographic and host‐related genetic and phenotypic differentiation. First, loci associated with eclosion time in Ragland et al. (2017) should show significant relationships with geographic variation in the population survey. Second, hawthorn fly populations at the more northern sites (Grant, MI, and Fennville, MI) should possess higher frequencies of alleles associated with earlier adult eclosion time in the GWAS than the more southern sites (Dowagiac, MI, and Urbana, Illinois [IL]) to track the generally earlier fruiting time of hawthorns further North (Figure 2a). Third, the apple race should mirror the geographic pattern exhibited by the hawthorn race (i.e., higher frequencies of alleles associated with earlier eclosion in northern than southern populations). Fourth, populations of apple flies, at both northern and southern sites, should possess higher frequencies of alleles related to earlier eclosion than their paired sympatric hawthorn population. Together, predictions three and four reflect that apple flies phenotypically eclose earlier than hawthorn flies at sympatric sites, but that both races eclose earlier at higher latitudes (Figure 3a).

## 2 MATERIALS AND METHODS

### 2.1 Geographic survey

Flies genotyped in the geographic survey were collected from nature as eggs or early instar larvae infesting apple or downy hawthorn fruit at four sympatric field sites across the Midwestern United States, where host trees and the fly races co‐occur within 1 km of each other (Figure 1). Collections were made from Grant, MI (lat., long. = 43.35 N, −85.9 W, year collected = 1989, *n* = 54 adult hawthorn and *n* = 48 adult apple flies genotyped, equally divided by sex), Fennville, MI (42.6 N, −86.15 W, 2008, *n* = 96 hawthorn and *n* = 93 apple flies), Dowagiac, MI (41.88 W, −86.23 N, 2006, *n* = 32 flies of each race), and Urbana, IL (40.08 W, −88.19 N, 2000, *n* = 48 hawthorn and *n* = 38 apple flies). Field‐collected flies were reared to adulthood for genotyping using standard *Rhagoletis* husbandry methods (see Feder, Chilcote, & Bush, 1989).

### 2.2 Genotyping by sequencing

Methods for genotyping by sequencing (GBS) of individually barcoded double‐digest restriction site‐associated DNA libraries of flies (ddRAD‐seq), de novo assembly of contigs, SNP calling, and allele frequency estimation for field‐collected samples at the four sites surveyed in the current study were performed as in Ragland et al. (2017). We used custom scripts and the Genome Analysis Toolkit (GATK version 2.5–2; DePristo et al., 2011) to identify 10,241 variable sites passing quality filters that were genotyped in the eclosion study and at all four paired field sites. Average SNP coverage per individual was 6.2X in the eclosion time GWAS and 3.3X in the geographic survey of sympatric sites.

### 2.3 Measuring linkage disequilibrium

To assess genome structure, Burrow's composite measures of linkage disequilibrium (∆) standardized to *r* values between −1 and 1 were estimated following Weir (1979) between pairs of SNPs within hawthorn and apple fly samples at the four collecting sites. Analysis of LD was restricted to a subset of 4,244 of the 10,241 total SNPs genotyped that were previously assigned to one of the five major chromosomes in the genome (Egan et al., 2015; Ragland et al., 2017). To further take LD and genome structure into account, we also separately analyzed three different classes of linked SNPs categorized as displaying high, intermediate, or low levels of LD within chromosomes, as defined in Ragland et al. (2017; see Supporting Information Appendix S1 for details). These high LD clusters of linked SNPs presumably represent inversions, with eight different groups identified on chromosome 2 and one each on the remaining four chromosomes.

### 2.4 Tests for allele frequency differences

Probabilities of single locus genotypes and allele frequencies for SNPs were calculated following McKenna et al. (2010). Tests for significant allele frequency differences for SNPs were performed using a nonparametric Monte Carlo approach between sample populations. We established a null distribution of allele frequency differences at each SNP for a given pair of samples (early‐ vs. late‐eclosing flies or flies from Grant vs. Urbana, constituting the latitudinal extremes of the sample) by generating 10,000 pairs of randomized samples. A pair of randomized samples consisted of *N* and *M* individuals (represented by their genotype probabilities) drawn with replacement from the pool of the two samples of respective sizes *N* and *M*. An observed absolute difference that exceeded the upper 95th quantile of the null Monte Carlo distribution of absolute differences was taken as significantly different than expected. To determine whether overall percentages of SNPs associated with eclosion time and geographic divergence were significantly greater than expected by chance, the total percentage of SNPs showing significant differences in each simulation was calculated and the distribution for all 10,000 runs compared to the observed percentages to assess whether these were in the upper 5% of simulated percentages. By using whole fly genotype probabilities to generate the null distribution, LD relationships among loci and, thus, the effects of inversions and genetic correlations between SNPs were accounted for by mirroring their associations in the randomized samples. Ragland et al. (2017) found that allele frequency differences between early‐ and late‐eclosing apple vs. hawthorn flies from Fennville were significantly correlated genome wide in the GWAS (*r* = 0.54, *p* < 0.0001 for all 10,241 SNPs genotyped). We therefore used the mean of the frequency difference averaged between the apple and hawthorn races for the allele associated with later eclosion time for all analyses involving eclosion time, except where noted.

### 2.5 Tests for genetic associations of eclosion time with geographic and host‐related differentiation

We performed linear regression analyses (R Core & Team, 2017) to assess the degree to which allele frequency differences for SNPs between the earliest and latest quantiles of flies in the eclosion time GWAS were related to (a) allele frequency differences within the apple and the hawthorn host races between the Grant and Urbana sites (i.e., a measure of the extent of geographic variation for flies between the two most distant sites we sampled, essentially encompassing the latitudinal range of overlap of the host races in the Midwest; see Figure 1) and (b) allele frequency differences between the apple and hawthorn races at the four sympatric sites (i.e., local host‐related divergence). Significance levels were determined by Monte Carlo simulations in which correlation coefficients were calculated between two random samples of whole fly genotype probabilities taken with replacement from the appropriate pooled data sets of flies, as above. Absolute values of observed correlation coefficients that exceeded the upper 5% of absolute r values in the simulated distributions of 10,000 replicates were taken as significantly different than expected. Again, by using whole fly genotype probabilities to generate null distributions, the nonindependence of SNPs displaying high LD with one another was factored into the analyses testing for significance. To visualize how overall patterns of SNP allele frequencies significantly associated with eclosion time changed geographically and between the races, we calculated mean standardized allele frequencies for apple and hawthorn fly populations at all sites. For eclosion time‐related SNPs (Ragland et al., 2017), we used the allele at a higher frequency in late‐eclosing flies as the reference, set the average allele frequency for each race across all sites to zero, and calculated the mean deviation in allele frequency for each population.

## 3 RESULTS

### 3.1 Linkage disequilibrium

**Figure 4:**
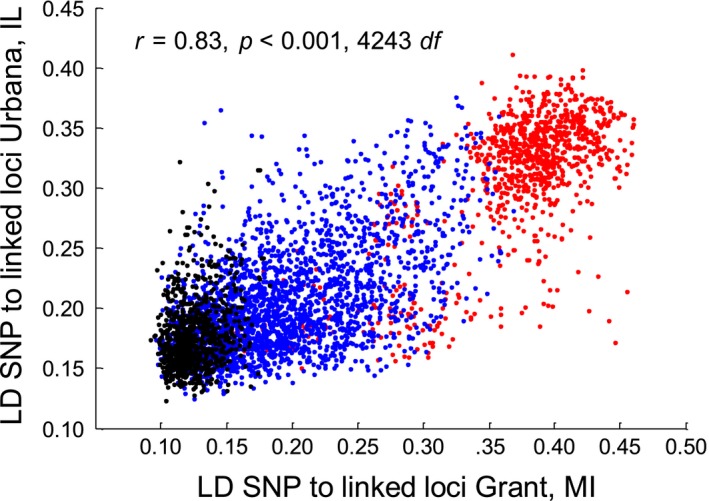
Relationship between linkage disequilibrium (LD) estimates made at Grant, MI, vs. Urbana, IL. Values are mean LD for a given SNP to all other SNPs mapping to the same chromosome in the apple and hawthorn races. SNPs belonging to different LD classes at the Grant site, as designated in Ragland et al. (2017), are depicted in different colors (red = high LD class of SNPs, blue = intermediate LD, black = low LD)

Patterns of composite LD between SNPs were similar within and across sites and between the host races. Mean pairwise LD values for SNPs to all loci mapping to the same chromosome were strongly correlated between populations from the ends of the transect at Grant and Urbana (*r* = 0.83, *p* < 0.0001, 4,243 *df*; Figure 4). Intrachromosomal mean pairwise LD values were also highly correlated between the host races at Grant (*r* = 0.90, *p* < 0.0001, 4,243 *df*) and Urbana (*r* = 0.79, *p* < 0.0001, 4,243 *df*). Likely due in large part to shared inversions and ongoing gene flow (Feder et al., 1994; Feder, Roethele, et al., 2003b), these similar patterns of LD imply that genome structure is concordant within and between the host races across the Midwest and allow for our broader application of the high, intermediate, and low LD SNPs classes categorized at Grant.

### 3.2 Patterns of geographic variation within and between the host races

Both hawthorn and apple host races displayed substantial geographic variation in SNP allele frequencies across sites. Of the 10,241 SNPs genotyped in the study, 2,871 (28%) showed significant allele frequency differences between hawthorn fly populations from Urbana (south) vs. Grant (north), while 2,102 (20.5%) differed for apple flies (*p* < 0.0001 and 0.001, respectively). Of these SNPs, 952 displayed significant geographic variation in both races. Thus, allele frequency differences between Grant and Urbana were significantly correlated between the host races (*r* = 0.49, *p* < 0.001, 10,240 *df*).

Two major trends in geographic variation were apparent. First, geographic divergence varied among chromosomes, being greater for SNPs on chromosomes 1–3 than on chromosomes 4 and 5 (Table 1). In an exception to this pattern, chromosome 1 showed less geographic variation in the apple vs. hawthorn race (Table 1). Conversely, chromosome 4 displayed increased differentiation in the apple vs. hawthorn race for high and intermediate LD loci. Second, geographic variation was higher for SNPs showing elevated levels of LD (Table 1). The percentages of SNPs displaying significant allele frequency differences between hawthorn fly populations from Grant and Urbana increased from the low (17%, *n* = 845), to intermediate (34.8%, *n* = 2,368) and to high LD (58.3%, *n* = 1,031) classes of loci (G‐heterogeneity test = 349.9, *p* < 0.0001, 2 *df*), although low LD SNPs on chromosomes 1–3 still showed significant geographic variation (Table 1). Together, these two trends support the first prediction of the standing variation hypothesis: High LD loci on chromosomes 1–3 displayed both the strongest associations with eclosion time (Ragland et al., 2017) and the highest levels of geographic divergence (Table 1).

**Table 1:** Percentages of SNPs displaying significant geographic allele frequency differences between Grant, MI, and Urbana, IL, for the apple race (upper table) and hawthorn race (lower table)

	chr 1	chr 2	chr 3	chr 4	chr 5	chr 1–5
Apple						
Map SNPs	*n* = 949	*n* = 675	*n* = 996	*n* = 436	*n* = 1,188	*n* = 4,244
	10.2 (0.06)	**38.9** (0.11)***	**43.1** (0.13)****	**29.1** (0.09)***	9.3 (0.06)	**24.2** (0.09)**
High LD	*n* = 263	*n* = 129	*n* = 223	*n* = 42	*n* = 374	*n* = 1,031
	7.2 (0.06)	**56.6** (0.14)**	**86.1** (0.24)****	**78.6** (0.17)**	6.7 (0.05)	**33.2** (0.11)**
Int. LD	*n* = 558	*n* = 459	*n* = 599	*n* = 159	*n* = 593	*n* = 2,368
	11.6 (0.06)	**38.6** (0.10)***	**35.2** (0.10)****	**40.3** (0.10)***	11.5 (0.06)	**24.7** (0.08)****
Low LD	*n* = 128	*n* = 87	*n* = 174	*n* = 235	*n* = 221	*n* = 845
	10.2 (0.06)	14.9 (0.07)	14.9 (0.07)	12.8 (0.06)	8.1 (0.06)	11.8 (0.06)
Hawthorn						
Map SNPs	*n* = 949	*n* = 675	*n* = 996	*n* = 436	*n* = 1,188	*n* = 4,244
	**54.4** (0.14)****	**50.4** (0.11)****	**47.7** (0.11)****	12.0 (0.06)	15.4 (0.06)	**37.0** (0.10)****
High LD	*n* = 263	*n* = 129	*n* = 223	*n* = 42	*n* = 374	*n* = 1,031
	**92.0** (0.25)****	**82.2** (0.16)****	**83.9** (0.20)***	4.8 (0.05)	17.1 (0.07)	**58.3** (0.15)****
Int. LD	*n* = 558	*n* = 459	*n* = 599	*n* = 159	*n* = 593	*n* = 2,368
	**45.5** (0.11)****	**47.1** (0.11)****	**42.1** (0.09)****	12.0 (0.06)	14.2 (0.06)	**34.8** (0.09)****
Low LD	*n* = 128	*n* = 87	*n* = 174	*n* = 235	*n* = 221	*n* = 845
	**15.6** (0.05)**	**20.7** (0.06)**	**20.7** (0.07)***	**14.9** (0.06)*	**15.8** (0.06)*	**17.0** (0.06)**

Notes

Results are given for all mapped SNPs (Map SNPs), and for high, intermediate (Int.), and low LD classes of SNPs for each chromosome considered separately, as well as for all chromosomes together (chr 1–5). Also given in parentheses are the mean SNP allele frequency differences between Grant, MI, and Urbana, IL, for apple and hawthorn fly populations for each class of SNP considered.

* *p* ≤ 0.05;

** *p* ≤ 0.01;

*** *p* ≤ 0.001;

**** *p* ≤ 0.0001; Significant percentages above null expectation for a class of SNPs, as determined by Monte Carlo simulations, are in bold. *n* = # SNPs genotyped in the class.

### 3.3 Eclosion time and geographic variation

**Figure 5:**
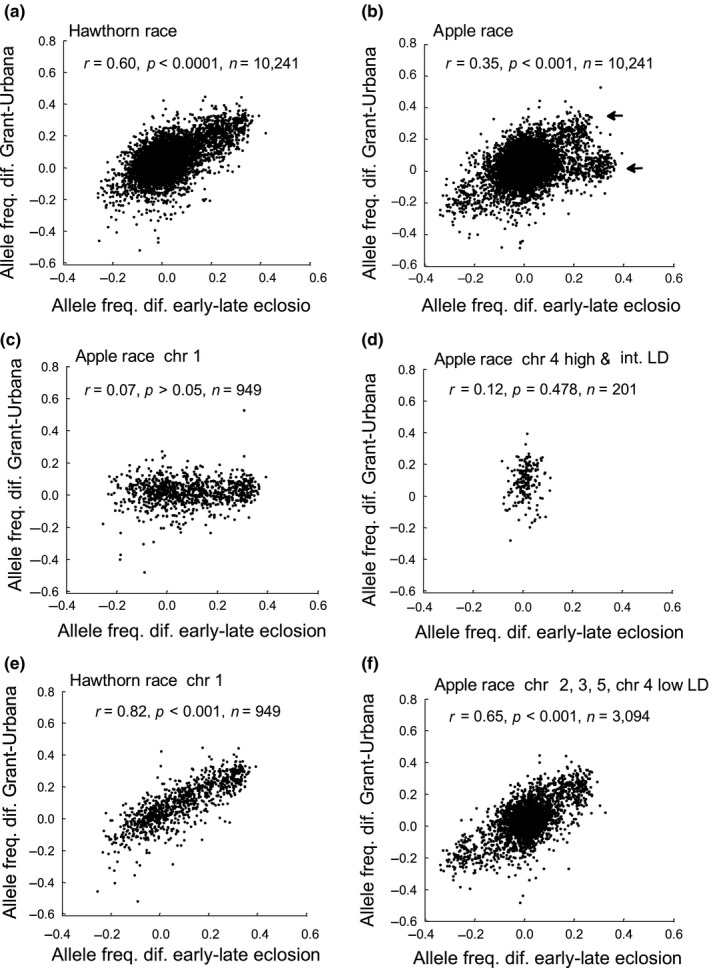
Relationships of the genetic responses of SNPs in the eclosion time GWAS (differences in allele frequencies for SNPs between early‐ and late‐eclosing quantiles of flies) vs. geographic divergence (differences in allele frequencies for SNPs between Grant and Urbana sites) for (a) all SNPs in the hawthorn race; (b) all SNPs in the apple race; (c) chromosome 1 SNPs in the apple race; (d) the high and intermediate classes of SNPs on chromosome 4 in the apple race; (e) chromosome 1 SNPs in the hawthorn race; and (f) chromosomes 2, 3, and 5, and the low LD class of loci on chromosome 4 in the apple race; *n* = number of SNPs in comparison. Arrows in panel b designate the dichotomous patterns of geographic variation displayed by the apple race. The lower arrow represents SNPs showing limited genetic association between eclosion time and geographic variation, while the upper arrow denotes SNPs displaying a more marked relationship

Allele frequency differences between the early‐ and late‐eclosing quantiles of flies in the GWAS of Ragland et al. (2017) were highly positively related to their latitudinal frequency differences from north to south between Grant and Urbana (Table 2). For all 10,241 SNPs, the genome‐wide correlation between the eclosion time GWAS and geographic differentiation in the hawthorn race was *r* = 0.60 (*p* < 0.0001; Figure 5a). The relationship was not as pronounced, but still significant, for the apple race (*r* = 0.35, *p* < 0.0001; Figure 5b).

**Table 2:** Correlation coefficients (*r*) of allele frequency differences for SNPs between the early‐ and late‐eclosing quantiles of flies in the eclosion time GWAS (Ragland et al., 2017) vs. geographic allele frequency differences within the host races between Grant, MI, and Urbana, IL, for apple fly (upper table) and hawthorn fly populations (lower table)

	chr 1	chr 2	chr 3	chr 4	chr 5	chr 1–5
Apple						
Map SNPs	*n* = 949	*n* = 675	*n* = 996	*n* = 436	*n* = 1,188	*n* = 4,244
	0.07	**0.72** ****	**0.72** ****	0.14	0.02	**0.42** ***
High LD	*n* = 263	*n* = 129	*n* = 223	*n* = 42	*n* = 374	*n* = 1,031
	**0.49** ****	**0.85** ****	**0.37** ***	0.02	0.06	**0.48** ***
Int. LD	*n* = 558	*n* = 459	*n* = 599	*n* = 159	*n* = 593	*n* = 2,368
	0.05	**0.65** ****	**0.63** ****	0.13	−0.01	**0.39** ****
Low LD	*n* = 128	*n* = 87	*n* = 174	*n* = 235	*n* = 221	*n* = 845
	−0.05	**0.43** **	0.15	0.03	−0.03	0.09
Hawthorn						
Map SNPs	*n* = 949	*n* = 675	*n* = 996	*n* = 436	*n* = 1,188	*n* = 4,244
	**0.82** ****	**0.73** ****	**0.69** ****	0.04	0.11	**0.72** ****
High LD	*n* = 263	*n* = 129	*n* = 223	*n* = 42	*n* = 374	*n* = 1,031
	**0.59** ****	**0.86** ****	**0.41** ****	0.17	−0.03	**0.84** ****
Int. LD	*n* = 558	*n* = 459	*n* = 599	*n* = 159	*n* = 593	*n* = 2,368
	**0.75** ****	**0.67** ****	**0.61** ****	−0.09	0.10	**0.63** ****
Low LD	*n* = 128	*n* = 87	*n* = 174	*n* = 235	*n* = 221	*n* = 845
	**0.37** ***	**0.44** **	0.19	0.10	0.01	**0.18** ***

Notes

Results are given for all mapped SNPs (Map SNPs), and for high, intermediate (Int.), and low LD classes of SNPs considered separately for each chromosome, as well as for all chromosomes together (chr 1–5). Significant relationships, as determined by Monte Carlo simulations, are in bold. *n* = # of SNPs genotyped in the class.

*p ≤ 0.05;

a **p ≤ 0.01;

b ***p ≤ 0.001;

c ****p ≤ 0.0001.

**Figure 6:**
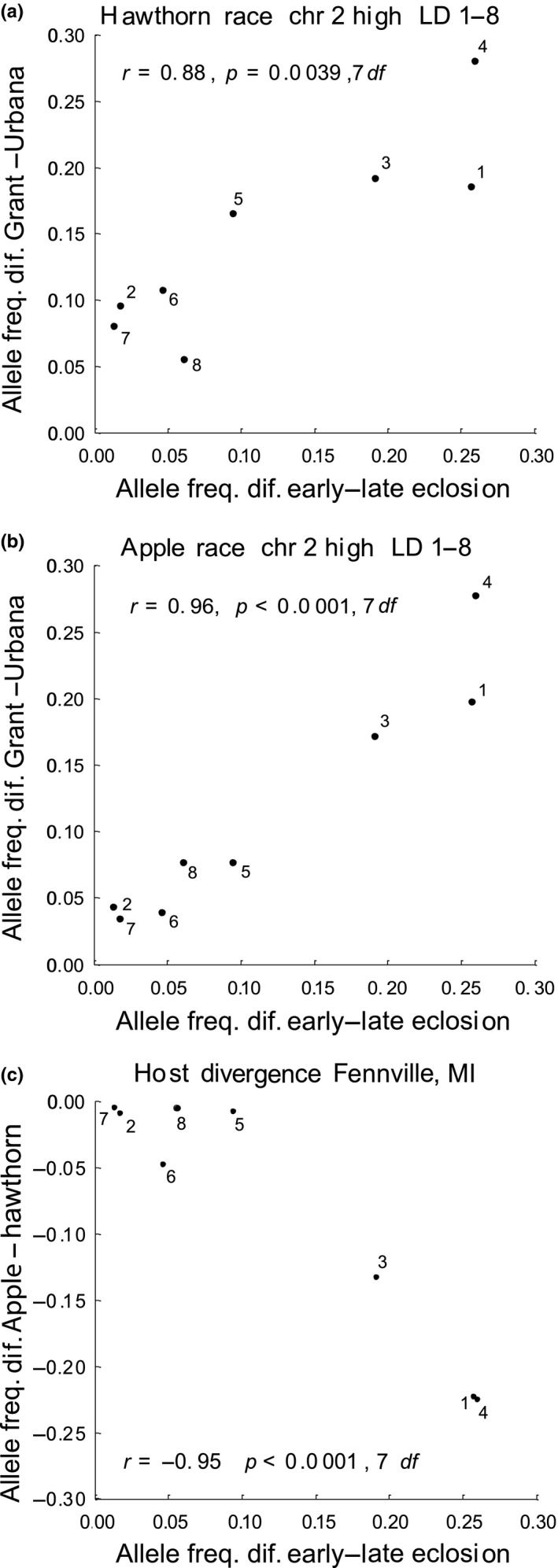
Relationships of the mean genetic responses of SNPs belonging to the eight different high LD groups on chromosome 2 in the eclosion time GWAS (i.e., their average allele frequency differences between early‐ and late‐eclosing quantiles of flies) vs. (a) their mean frequency differences in the hawthorn race between Grant and Urbana; (b) their mean frequency differences in the apple race between Grant and Urbana; and (c) their mean frequency differences between the apple and hawthorn races at Fennville, MI. Numbers (1–8) in figures designate the eight different groups of high LD loci, presumably representing eight different relatively small‐sized inversions residing on chromosome 2

For the hawthorn race, the genetic relationship between eclosion time and geographic SNP variation was significant for chromosomes 1–3 (*r* = 0.78, *p* < 0.0001, *n* = 2,620 SNPs) but not for the remainder of the genome (chromosomes 4 and 5, *r* = 0.09, *p* = 0.396, *n* = 1,624 SNPs; Table 2). The genetic correlation between eclosion time and geographic variation was also high among the eight different groups of high LD SNPs on chromosome 2 that presumably represent smaller inversions (*r* = 0.88, *p* = 0.0039, 7 *df*; Figure 6a). Consistent with the second prediction of the standing variation hypothesis, the allele that increased in frequency in the hawthorn race toward the northernmost Grant vs. southernmost Urbana site was generally the allele found in higher frequency in the early‐eclosing quantile of flies in the GWAS. Specifically, this relationship was observed for 78.3% of the 2,196 SNPs showing a significant association with eclosion time and 90.1% for the subset of 1,376 of these SNPs mapping to chromosomes 1–3 (*p* < 0.0001 in both cases, as determined by Monte Carlo simulations).

The weaker relationship between eclosion time and geographic SNP variation in the apple race reflected differences among chromosomes and LD classes. For the apple race, Figure 5b suggests that SNPs can be divided into two categories: (a) loci displaying the same positive relationship between eclosion and geographic variation seen in the hawthorn race (upper arrow in Figure 5b) and (b) loci that do not (lower arrow). This second category in apple flies was largely comprised of SNPs mapping to chromosome 1 and to the high and intermediate LD classes on chromosome 4 (Table 1). There was no significant relationship between eclosion time and geographic SNP variation for apple flies for chromosome 1 (*r* = 0.07, *p* = 0.615, *n* = 949 SNPs; Figure 5c) or for the high and intermediate LD classes of SNPs on chromosome 4 (*r* = 0.12, *p* = 0.478, *n* = 201 SNPs; Figure 5d), although such a relationship clearly was seen, at least for chromosome 1, in the hawthorn race (*r* = 0.82, *p* = <0.001, *n* = 949 SNPs; Figure 5e). Without chromosome 1 and the high and intermediate LD SNPs on chromosome 4, the dichotomy seen for apple flies disappeared (Figure 5f) and the correlation coefficient between eclosion time and geographic SNP variation increased in the apple race from *r* = 0.35 to 0.65 (*p* < 0.0001, *n* = 3,094 SNPs). Moreover, like the hawthorn race, there was a strong relationship in the apple race between eclosion time and geographic variation for the eight groups of high LD SNPs on chromosome 2 (*r* = 0.96, *p* < 0.0001, 7 *df*; Figure 6b). Also similar to hawthorn flies, the allele in higher frequency in the apple race at the Grant compared to the Urbana site was the allele at higher frequency in the early‐eclosing quantile of flies in the GWAS, consistent with prediction three of the standing variation hypothesis. Even with chromosomes 1 and 4 included, 69.7% of the 2,196 SNPs showing a significant association with eclosion time changed frequency in the expected direction in the apple race, toward early eclosion alleles at Grant, while this relationship was observed for 88.7% of these SNPs (*n* = 1,672) mapping to chromosomes 2–3 (*p* < 0.0001 in both cases).

### 3.4 Host race, eclosion, and geographic differentiation across the genome

Genomic variation associated with eclosion time was also significantly related to host race divergence at the Grant, Fennville, Dowagiac, and Urbana sites. However, this relationship varied across chromosomes and was dependent upon the pattern of geographic variation displayed by each LD class (Table 3). In particular, host race divergence at sympatric sites was significantly associated with eclosion time for chromosomes 1–3 (Table 3). In contrast, no significant relationship was detected for chromosomes 4 or 5 at any site (Table 3). We therefore focus on chromosomes 1–3 below.

**Table 3:** Correlation coefficients (*r*) of allele frequency differences for SNPs between early‐ and late‐eclosing quantiles of flies in the eclosion time GWAS (Ragland et al., 2017) vs. host race allele frequency differences between apple and hawthorn flies where populations co‐occur at field sites in Grant, MI, Fennville, MI, Dowagiac, MI, and Urbana, IL

	chr 1	chr 2	chr 3	chr 4	chr 5	chr 1–5
Grant, MI						
Map SNPs	*−0.64* **	−0.16	*−0.46* **	0.08	0.02	*−0.42* ***
High LD	−0.08	*−0.52* ****	−0.09	−0.14	0.07	*−0.63* ***
Int. LD	*−0.51* ***	−0.08	*−0.40* **	0.20	0.04	*−0.29* ***
Low LD	*−0.25* *	0.05	−0.04	−0.03	0.03	−0.06
Fennville, MI						
Map SNPs	*−0.75* ****	*−0.80* ****	−0.14	0.18	−0.16	*−0.56* ****
High LD	*−0.43* ****	*−0.92* ****	−0.19	−0.20	0.02	*−0.66* ****
Int. LD	*−0.66* ****	*−0.75* ****	−0.07	0.15	−0.17	*−0.50* ****
Low LD	*−0.28* **	*−0.47* ***	−0.14	0.12	0.01	*−0.12* *
Dowagiac, MI						
Map SNPs	***0.45*** *	*−0.29* **	*−0.48* **	0.07	−0.02	−0.01
High LD	0.10	*−0.33* **	−0.15	0.28	−0.12	0.01
Int. LD	***0.42*** **	*−0.33* **	*−0.36* **	0.09	−0.01	−0.04
Low LD	***0.23*** *	−0.18	*−0.23* *	0.01	−0.01	−0.03
Urbana, IL						
Map SNPs	***0.71*** ****	−0.06	*−0.52* **	−0.09	0.11	***0.18*** *
High LD	***0.30*** **	−0.11	−0.02	0.06	−0.05	0.20
Int. LD	***0.62*** ****	0.03	*−0.47* **	−0.11	0.13	***0.13*** *
Low LD	***0.25*** *	0.03	−0.10	0.04	0.07	0.03

Notes

Results are given for all mapped SNPs (Map SNPs), and for high, intermediate (Int.), and low LD classes of SNPs for each chromosome considered separately, as well as for all chromosomes together (chr 1–5). Significant relationships positive in sign are highlighted in bold, and negative relationships are in italics. See Tables 1 and 2 for # of SNPs genotyped in each class.

* *p* ≤ 0.01;

** *p* ≤ 0.01;

*** *p* ≤ 0.001;

**** *p* ≤ 0.0001.

Patterns of host‐related allele frequency divergence were similar between chromosomes 2 and 3, but differed for chromosome 1. When considered together, chromosomes 2 and 3 displayed a consistent pattern of divergence in mean eclosion‐standardized allele frequencies between apple and hawthorn host races across sympatric sites (Figure 3b; see Section 2 for details of eclosion standardization calculations). Moreover, in accord with the third prediction of the standing variation hypothesis, mean standardized allele frequencies for chromosomes 2 and 3 showed a parallel decrease with increasing latitude for both host races, with southern populations of both apple and hawthorn flies possessing higher frequencies of alleles associated with later eclosion in the GWAS (Figure 3b). However, while eclosion time was significantly related to host race divergence at each of the four sympatric sites for chromosomes 2 and 3 (*r* = −0.36, −0.55, −0.43, and −0.38, for Grant, Fennville, Dowagiac, and Urbana, respectively; *p* < 0.0001 in all cases, *n* = 1,671 SNPs), the signs (directions) of the relationships were all negative. This pattern extended to the eight high LD groups of SNPs on chromosome 2, as well (Figure 6c). Thus, in contrast to the fourth prediction of the standing variation hypothesis, apple fly populations tended to possess higher frequencies of alleles associated with later adult eclosion at all four sites (Figure 3b), even though apple flies eclose, on average, earlier than sympatric hawthorn flies (Figure 3a).

The different pattern displayed by chromosome 1 may partially counteract this deviation from prediction four (Figure 3c). For SNPs on chromosome 1, geographic variation was less pronounced in the apple than hawthorn race (Table 1) and was not significantly correlated with eclosion time for apple flies (Figure 5c), as it was for hawthorn flies (Figure 5e). This was true even though a significant percentage of SNPs on chromosome 1 were associated with eclosion time (41.4% = 393/949 SNPs; *p* < 0.0001) and the associations (allele frequency difference between early‐ and late‐eclosing flies) were significant and positively correlated between apple and hawthorn flies (*r* = 0.88, *p* < 0.0001, *n* = 949 SNPs). As a consequence of the reduced geographic variation in the apple race, mean standardized allele frequency clines for chromosome 1 crossed between the host races, with apple fly populations at Urbana and Dowagiac tending to have the expected lower frequencies of alleles associated with later adult eclosion, while the reverse was true at Grant and Fennville (Figure 3c). As a result, correlations between eclosion time and host‐related divergence were significant and positive in sign at Urbana and Dowagiac, in line with prediction four, while they were significant and negative in sign at Grant and Fennville (Table 3). When chromosomes 1–3 were considered collectively, the effects of chromosome 1 outweighed those of chromosomes 2 and 3, such that standardized allele frequencies correctly predicted that apple flies should eclose earlier than hawthorn flies at Urbana and Dowagiac (Figure 3d). Moreover, mean standardized allele frequencies predicted that the apple fly population at Dowagiac would deviate from the other sites and eclose relatively late (Figure 3d), as observed in nature (Figure 3a). Nevertheless, mean standardized allele frequencies for chromosomes 1–3 still predicted that apple flies at Grant and Fennville would eclose later than hawthorn flies (Figure 3d), deviating from the observed phenotypic pattern (Figure 3a) and running counter to prediction four of the standing variation hypothesis.

## 4 DISCUSSION

### 4.1 Standing variation and rapid adaptive evolution

Our results support the importance of standing variation in the recent formation of the apple‐infesting host race of *R. pomonella*, a prime example of ecological divergence with gene flow (Berlocher & Feder, 2002). The patterns of eclosion‐associated genomic divergence observed in the population survey of the Midwest largely concurred with the predictions of the standing variation hypothesis, with one major exception. First, geographic and host‐related divergence was most pronounced for chromosomes 1–3, particularly for the high LD loci, reflecting the pattern of eclosion‐associated variation reported by Ragland et al. (2017). Second, clinal variation within the ancestral hawthorn host race tracked geographic variation in hawthorn fruiting time (Figure 3a). Hawthorn fly populations at the more northern Grant and Fennville sites possessed higher frequencies of alleles associated with early adult eclosion, providing a potential seed for the shift to the earlier fruiting apple. Third, the apple race generally mirrored the geographic pattern exhibited by the hawthorn race, although in this regard there was an important difference we discuss further below. Finally, chromosome 1 loci associated with early eclosion were found at higher frequencies in apple vs. hawthorn flies at the two southern sites (Dowagiac and Urbana), accounting for the earlier eclosion of apple flies. However, important questions regarding the earlier eclosion of apple flies at the northern Grant and Fennville sites remain, as apple flies at these sites tended to have higher frequencies of late eclosion alleles, compared to sympatric hawthorn flies.

Previous work has demonstrated that eclosion time is associated with many SNPs of modest effect spread throughout chromosomes 1–3 (Ragland et al., 2017), which is inconsistent with a recent mutational origin for the genetic basis of the shift to apples. The associations of SNP alleles with eclosion time and their genetic architecture, as implied by patterns of LD, are similar between the races. In particular, the same sets of alleles, in similar linkage phase on chromosomes 1–3, appear to affect eclosion time in a concordant manner both locally and globally across the Midwest, within and between the host races. These observations, combined with the recent time frame of divergence (~400 years since the introduction of apples to North America), argue strongly against multiple independent origins of new mutations underlying the genetics of eclosion time. It is highly unlikely that the same variants have arisen and diverged between host races in a similar manner repeatedly across sites and on such a rapid timescale.

Future studies are needed to identify the specific genes affecting eclosion time in *R. pomonella*, test for signatures of selection, and discern their demographic histories. In this regard, Ragland, Egan, Feder, Berlocher, and Hahn (2011) and Meyers et al. (2016) showed marked differences between host races in gene expression patterns prior to diapause termination and suggested genes in the Wnt and TOR signaling pathways as candidate loci. Whole‐genome DNA sequencing is also needed examine the apple race for soft vs. hard selective sweeps around candidate loci (Hermisson & Pennings, 2005), the former predicted under the standing variation hypothesis, while the latter is expected if new mutations enabled the shift to apple. In addition, previous analysis of cDNA loci implied that the putative inversions on chromosomes 1–3 containing eclosion loci are old (>1.5 Mya; Feder, Berlocher, et al., 2003; Feder et al., 2005). Thus, as discussed earlier, if secondary contact and gene flow with Mexican flies from the EVTM generated the standing variation in the hawthorn race, then we predict that genome sequencing studies will reveal that many alleles affecting eclosion will date to ~1.5 Mya.

However, this does not mean that variants of large effect (Colosimo et al., 2005; Hopkins & Rausher, 2011, 2012; Lamichhaney et al., 2015; Steiner et al., 2007; Yuan, Sagawa, Young, Christensen, & Bradshaw, 2013) and of recent mutational origin (e.g., industrial melanism in the peppered moth; van’t Hof, 2016) do not also play important roles in ecological adaptation and speciation. This could even be the case for *Rhagoletis* with respect to loci affecting other selected traits such as host plant choice (Dambroski et al., 2005). Future work is needed to establish the genomic architecture of host odor preference in *R. pomonella* and infer the role of new mutations vs. standing variation in its contribution to host race divergence.

### 4.2 Eclosion time and genomic divergence between the host races

Notably, our results differed from prediction four of the standing variation hypothesis: On balance, apple flies did not have higher frequencies of alleles associated with earlier eclosion than sympatric hawthorn flies. Although we found substantial standing geographic genetic variation for eclosion time on chromosomes 1–3 in the hawthorn race, which was significantly correlated with host‐related divergence at all sympatric sites, the direction of the allele frequency differences between the races generally differed from expectation. Frequencies of chromosome 2 and 3 SNPs changed in parallel across all sites in both apple and hawthorn host races, but showed a paradoxical pattern within each sympatric site, where apple flies tended to possess higher frequencies of alleles associated with later eclosion than hawthorn flies (Table 3 and Figure 3d). However, clinal patterns for chromosome 1 SNPs deviated from prediction three, showing a steep cline among hawthorn fly populations, but remaining relatively flat among apple fly populations. Therefore, chromosome 1 SNPs actually fit the prediction four pattern at Dowagiac and Urbana with apple flies harboring higher frequencies of alleles associated with earlier eclosion, although they still showed the reversed pattern at Grant and Fennville.

One possible reason for this discrepancy may be that alleles affecting or affected by eclosion time are in different linkage phases between the host races at Grant and Fennville vs. the two more southern sites. However, patterns of LD between SNPs were similar across geographic sites and between the host races at sympatric sites (Figure 4). Moreover, SNPs on chromosomes 1–3 were significantly correlated in their effects on eclosion time between the host races (*r* = 0.74, *p* < 0.0001, *n* = 2,620 SNPs). Thus, the earlier eclosion phenotype of apple flies cannot be easily accounted for by geographic or host‐associated variation in linkage phase between genes affecting eclosion time and the chromosome 1–3 alleles genotyped in the current study. Additional eclosion time studies involving whole‐genome sequencing at Fennville and the other sites are still needed, however, to completely discount the scenario that variable linkage relationships between the host races are responsible for the earlier eclosion time of apple flies.

Another important consideration is that we may not have genotyped all relevant eclosion‐associated SNPs, including key loci responsible for the earlier eclosion of apple vs. hawthorn flies at Grant and Fennville. However, a relatively high percentage of the phenotypic variation in eclosion time (63%) was explained by the 10,241 SNPs we genotyped (Ragland et al., 2017). In addition, although many SNPs (*n* = 213) displayed significant allele frequency differences between early‐ and late‐eclosing flies only in the apple race GWAS (Ragland et al., 2017), these loci showed no significant genetic relationship between eclosion time and geographic variation in the apple race (*r* = 0.004, *p* = 0.972) or with host‐related divergence at any site (*r* = 0.02, 0.05, 0.10, and 0.01 for Grant, Fennville, Dowagiac, and Urbana, respectively; *p* > 0.50 in all cases). Thus, SNPs with eclosion time effects unique to apple flies cannot readily explain why apple flies eclose earlier than hawthorn flies at Grant and Fennville. Nevertheless, the effects of loci not genotyped in the study, which are in linkage equilibrium (low LD) with the 10,241 SNPs we scored, may remain undetected and thus a possible cause of earlier apple fly eclosion. These unresolved loci could conceivably be of large effect and of recent mutational origin. However, they may also have relatively small effect sizes and represent older standing variation, which in combination with residence in freely recombining genomic regions, could further account for their absence from the current study. Additional whole‐genome sequencing is needed to resolve the issue of undetected loci.

Finally, gene‐by‐environment or nonadditive epistatic interactions may also play a role in determining eclosion time (Filchak, Roethele, & Feder, 2000). In the former case, differences in the pre‐ or overwintering period experienced by the host races at the more northern Grant and Fennville sites may result in alleles associated with later diapause termination in the laboratory having different effects in nature, causing apple flies to eclose earlier (Filchak et al., 2000). In insects, most latitudinal clines associated with diapause vary in response to photoperiod (Hut, Paolucci, Dor, Kyriacou, & Daan, 2013), including voltinism clines in the European corn borer that contribute to temporal isolation between pheromone strains (Levy, Kozak, & Dopman, 2018; Levy, Kozak, Wadsworth, Coates, & Dopman, 2015). Photoperiod also affects diapause in *Rhagoletis* (Prokopy, 1968). Thus, it is possible that longer photoperiods experienced by larval apple flies or by their mothers may cause them to eclose earlier than hawthorn flies in a manner not predicted by their genotypes. However, temperature and the length of the prewinter period following pupariation have been shown to exert a larger influence than photoperiod on eclosion phenotype in *R. pomonella* (Filchak, Roethele, & Feder, 2001). Moreover, Dambroski and Feder (2007) controlled for photoperiod and verified that there is a genetic basis for apple flies eclosing earlier than hawthorn flies, including at the Grant site. With respect to epistasis, it is possible that interactions between SNPs on chromosomes 1–3 might cause apple flies to eclose earlier than hawthorn flies. Elevated LD between these loci might be expected if strong epistatic interactions between chromosomes influenced the trait. However, considering the host races together, we did not find strong LD between eclosion‐associated SNPs residing on different chromosomes (data not shown). Further study is needed to resolve the genetic basis for the earlier eclosion of apple flies at the Grant and Fennville sites, although we consider undetected loci as perhaps the most likely explanation at the current time.

It also remains to be determined why apple flies at Dowagiac eclose relatively late in controlled laboratory rearing experiments (Figure 3a) and as predicted genetically (Figure 3d), yet in nature they remain diverged from hawthorn flies for chromosome 1–3 alleles (Figure 3b,c). Apples collected at Dowagiac fruited relatively late in the season and at a more similar time to hawthorns (see site 3 in Figure 2a,c), which may contribute to the greater phenotypic similarity in eclosion time between Dowagiac apple and hawthorn flies relative to other sites (Figure 3a). Thus, allochronic isolation should be reduced at Dowagiac but apple flies still maintain significant allele frequency differences for SNPs associated with eclosion time there (Figure 3b,c). Additional field‐based host fidelity studies and GWAS for other traits, such as host odor preference, are needed to fully characterize differentiation between host races at this site.

### 4.3 Geographic variation not explained by eclosion time

Our results also raise questions concerning why genomic regions, other than those harboring eclosion variation (chromosomes 1–3), display significant geographic variation. For example, the high and intermediate LD classes of SNPs on chromosome 4 show significant geographic variation in the apple race (Table 1) but are not associated with eclosion time (Table 2). The same is true in the hawthorn race for the low LD class of SNPs on chromosomes 4 and 5 (Tables 1 and 2). These results suggest that other factors, in addition to eclosion time, impose differential selection across the Midwest. In this regard, previous studies imply that the length of the prewinter period also differentially selects on the intensity at which flies initially enter pupal diapause. (Egan et al., 2015; Feder, Roethele, Wlazlo, & Berlocher, 1997). The prewinter period is longer for apple than hawthorn flies and also varies with latitude (lengthening from north to south), resulting in both geographic and host‐related differential selection on initial diapause intensity (Dambroski & Feder, 2007). Moreover, initial diapause intensity may be under separate genetic control from eclosion time, at least in hawthorn flies (Ragland et al., 2017). Thus, selection on other aspects of the diapause phenotype might account for the significant geographic variation not explained by eclosion time.

## 5 CONCLUSION

In conclusion, we have shown that selection on eclosion time variation significantly sculpts geographic and host‐related genomic differentiation in the apple and hawthorn races of *R. pomonella*. Diapause life history adaptation in the recently formed apple race appears to have been extracted, in significant part, from standing genetic variation in the ancestral hawthorn race. Thus, our results highlight the potential inherent in geographic clines to contribute to the origins of new biodiversity when new resource opportunities become available. However, details concerning how this process has resulted in apple flies eclosing earlier than hawthorn flies, particularly at the more northern Grant and Fennville sites, remain to be resolved. Regardless, in *Rhagoletis*, polygenic standing variation, particularly in putative inverted regions, appears to be due to a history of geographic isolation and secondary contact (Feder et al., 2005; Feder, Berlocher, et al., 2003). How general this is for other model systems of ecological speciation remains to be determined. Similarly, whether selection has widespread genomic effects on divergence in other organisms, as it does in *Rhagoletis*, or has simpler genetic underpinnings and consequences for facilitating speciation, is an open question. Nevertheless, it is clear that intraspecific clinal variation in life history timing is extensive in other organisms, including plants (Blackman, Michaels, & Rieseberg, 2011; Chen et al., 2012; Kawkakami et al., 2011; Keller, Levsen, Olson, & Tiffin, 2012; Lowry & Willis, 2010), vertebrates (Johnsen et al., 2007; O’Malley, Ford, & Hard, 2010), and insects (Adrion, Hahn, & Cooper, 2015; Bradford & Roff, 1995; Bradshaw, Quebodeaux, & Holzapfel, 2003; Kyriacou, Peixoto, Sandrelli, Costa, & Tauber, 2008; Lankinen, Tyukmaeva, & Hoikkala, 2013; Lehmann, Lyytinen, Piiroinen, & Lindström, 2015; Levy et al., 2015; Masaki, 1978; Paolucci, Zande, & Beukeboom, 2013; Roff, 1980; Wang et al., 2012). However, whether and how this intraspecific clinal variation is transformed into interspecific divergence are less well documented. It would seem that divergent selection on standing variation in life history timing could be a prime axis for contributing to the adaptation of organisms to new habitats and environments (review by Taylor & Friesen, 2017), potentially generating reproductive isolation and a wealth of new biodiversity.

## CONFLICTS OF INTEREST

All authors confirm that they have no conflicts of interest.

## AUTHOR CONTRIBUTIONS

MMD, SPE, GJR, PJM, GRH, THQP, DAH, SHB, PN, and JFL developed the conceptual framework and experimental design; SPE, PJM, GRH, and THQP collected the data; MMD, GJR, PL, and JLF performed analyses; and all authors contributed to manuscript drafting.

## DATA ACCESSIBILITY

Data from the eclosion GWAS were previously deposited in DRYAD (https://doi.org/10.5061/dryad.kn568). All new sequence data generated for this study are deposited in DRYAD (https://doi.org/dryad.k42t7g2).
