# Supplementary material for: Standing geographic variation in eclosion time and the genomics of host race formation in Rhagoletis pomonella fruit flies
Source: Ecol Evol. 2018 Dec 14;9(1):393–409. doi: 10.1002/ece3.4758 (PMC6342182; doi:10.1002/ece3.4758)
Supplement: Supplementary file 1 [file ECE3-9-393-s001.docx]

**Table S1.** Percentages of SNPs showing significant allele frequency differences between the earliest <3% and latest >97% eclosing quantiles of individuals considering hawthorn and apple flies together in the GWAS of Ragland et al. (2017) for all mapped SNPs, and for high, intermediate (Int.), and low LD classes of SNPs for each chromosome separately, as well as all together (chr 1-5). Significance levels are indicated where the percentage of SNPs in a category exceeded that expected by chance, as determined by Monte Carlo sampling of whole individual genotypes: * = P < 0.01; ** = P < 0.01; *** = P < 0.001; ****P = < 0.0001; significant results are highlighted in grey boxes. Mean absolute allele frequency differences for SNPs between early and late eclosing flies for each SNP class are also given in parentheses. n = # of SNPs

genotyped in the class.

| Ecl. time | chr 1 | chr 2 | chr 3 | chr 4 | chr 5 | chr1-5 |
| --- | --- | --- | --- | --- | --- | --- |
|  |  |  |  |  |  |  |
| All SNPs | n = 949 | n = 675 | n = 996 | n = 436 | n = 1188 | n = 4244 |
|  | 63.7^****^ | 47.4^****^ | 45.2^****^ | 4.6 | 4.5 | 34.2^****^ |
|  | (0.14) | (0.10) | (0.09) | (0.03) | (0.03) | (0.08) |
| High LD | n = 263 | n = 129 | n = 223 | n = 42 | n = 374 | n = 1031 |
|  | 95.1^****^ | 68.2^****^ | 90.1^****^ | 4.8 | 0.5 | 52.7^****^ |
|  | (0.25) | (0.16) | (0.18) | (0.03) | (0.03) | (0.13) |
| Int. LD | n = 558 | n = 459 | n = 599 | n = 159 | n = 593 | n = 2368 |
|  | 59.3^****^ | 47.9^****^ | 37.1^****^ | 2.5 | 6.4 | 34.4^****^ |
|  | (0.10) | (0.09) | (0.07) | (0.03) | (0.04) | (0.07) |
| Low LD | n = 128 | n = 87 | n = 174 | n = 235 | n = 221 | n = 845 |
|  | 18.8^***^ | 13.8^*^ | 16.9^***^ | 5.9 | 6.3 | 10.9^**^ |
|  | (0.05) | (0.04) | (0.05) | (0.03) | (0.03) | (0.04) |
