# Supplementary material for: Standing geographic variation in eclosion time and the genomics of host race formation in Rhagoletis pomonella fruit flies
Source: Ecol Evol. 2018 Dec 14;9(1):393–409. doi: 10.1002/ece3.4758 (PMC6342182; doi:10.1002/ece3.4758)
Supplement: Supplementary file 2 [file ECE3-9-393-s002.docx]

**Table S2.** Numerical designations (Pop.) for 18 different *Rhagoletis pomonella* populations at 13 sites appearing in Figures 1-3, constituting a north to south transect from Grant, MI, USA to Chiapas, Mexico. Given are the literature references (Ref.) for data, locations of sites, host plant species of origin for fly populations, latitude [N] and longitude [W] in degrees, date and year flies were sampled, numbers of individuals (n), and mean number of days to eclosion (+ s.e.) of populations in controlled rearing experiments for flies collected from the sites. See Figure 1 for a map of sites and ranges of flies. Reference 1 = Hood et al. (2015); Reference 2 = Lyons-Sobaski and Berlocher (2009); Reference 3 = Xie et al. (2007); Reference 4 = Dambroski and Feder (2007); see Supplementary Information or Main Text for full citations. Sites 1-4 = Midwestern, USA; sites 5-8 = state of Texas, USA; sites 9, 10 = Sierra Madre Oriental Mountains, MX (SMO); sites 11, 12 = Eje Volcánico Trans Mexicano region of central Mexico (EVTM); site 13 = Chiapas Highlands, Mexico.

| Pop. | Ref. | Location | Host plant species of origin | Lat., Long. | Date | n | Mean days eclosion (s.e.) |
| --- | --- | --- | --- | --- | --- | --- | --- |
| 1H | 1 | Grant, Newaygo Co., MI, USA | *Crataegus* *mollis* | 43.35, -85.90 | 09/16/02 | 219 | 65.0 days (+ 0.9) |
| 1A | 1 | Grant, Newaygo Co., MI, USA | *Malus domestica* | 43.35, -85.90 | 08/06/02 | 709 | 56.5 days (+ 0.4) |
| 2H | 1 | Fennville, Allegan Co., MI | *Crataegus* *mollis* | 42.60, -86.15 | 09/15/02 | 1644 | 65.1 days (+ 0.9) |
| 2A | 1 | Fennville, Allegan Co., MI | *Malus domestica* | 42.60, -86.15 | 08/05/02 | 1000 | 58.5 days (+ 0.3) |
| 3H | 1 | Dowagiac, Cass Co., MI | *Crataegus* *mollis* | 41.88, -86.23 | 09/17/06 | 528 | 74.5 days (+ 0.8) |
| 3A | 1 | Dowagiac, Cass Co., MI | *Malus domestica* | 41.88, -86.23 | 09/08/02 | 1598 | 73.7 days (+ 0.4) |
| 4H | 1 | Urbana, Champaign Co., IL, USA | *Crataegus* *mollis* | 40.08, -88.19 | 09/21/02 | 47 | 75.7 days (+ 2.6) |
| 4A | 1 | Urbana, Champaign Co., IL, USA | *Malus domestica* | 40.08, -88.19 | 08/06/99 | 111 | 64.4 days (+ 1.8) |
| 5 | 2 | Waxahachie, Ellis Co., TX, USA | *Crataegus* *mollis v. texana* | 32.23, -96.50 | 10/06/89 | 75 | 120.3 days (+ 1.0) |
| 6B | 2 | Urbana, San Jacinto Co., TX, USA | *Crataegus* *brachyacantha* | 30.33, -95.11 | 09/06/89 | 26 | 87.9 days (+ 4.6) |
| 6V | 2 | Urbana, San Jacinto Co., TX, USA | *Crataegus* *viridis* | 30.33, -95.11 | 10/29/89 | 270 | 104.9 days (+ 2.0) |
| 7 | 2 | Tivydale, Gillespie Co., TX, USA | *Crataegus* *greggiana* | 30.14, -99.11 | 10/08/89 | 39 | 126.0 days (+ 2.6) |
| 8 | 4 | Brazos Bend, Ft. Bend Co., TX, USA | *Crataegus* *viridis* | 29.24, -95.37 | 10/17/00 | 38 | 126.5 days (+ 6.1) |
| 9 | 3 | San Joaquin, Queretaro, Mexico | *Crataegus* *rosei parrayana* | 20.55, -99.34 | 08/24/02 | 106 | 93.7 days (+ 1.1) |
| 10 | 3 | Casa Blanca, Veracruz, Mexico | *Crataegus* *mexicana* | 19.38, -97.06 | 09/24/02 | 88 | 83.5 days (+ 0.9) |
| 11 | 3 | Tancitaro, Michoacan, Mexico | *Crataegus* *mexicana* | 19.20, -102.22 | 11/15/02 | 8 | 151.2 days (+ 5.5) |
| 12 | 3 | Coajomulco, Morelos, Mexico | *Crataegus* *mexicana* | 19.03, -99.11 | 11/12/02 | 41 | 151.5 days (+ 2.3) |
| 13 | 3 | San Cristobal, Chiapas, Mexico | *Crataegus* *mexicana* | 16.45, -92.38 | 11/15/02 | 36 | 133.9 days (+ 0.7) |
