# Supplementary material for: Standing geographic variation in eclosion time and the genomics of host race formation in Rhagoletis pomonella fruit flies
Source: Ecol Evol. 2018 Dec 14;9(1):393–409. doi: 10.1002/ece3.4758 (PMC6342182; doi:10.1002/ece3.4758)
Supplement: Supplementary file 3 [file ECE3-9-393-s003.docx]

**Supplementary Information**

**Eclosion time GWAS**

In Ragland et al. (2017), samples of apple and hawthorn flies collected as larvae feeding in infested fruit at the Fennville, MI site in 2009 were transported back to the laboratory and reared to adulthood using standard *Rhagoletis* husbandry methods (see Egan et al. 2015; Ragland et al. 2017). After overwintering, petri dishes containing fly pupae were placed in a 21°C temperature-controlled room and monitored daily for newly eclosing adults. The date of eclosion and sex of each individual were recorded and the fly subsequently frozen at -80°C for later genetic analysis (total n = 1,250 eclosing apple fly and 1,644 hawthorn fly adults). The 48 (≤ 3% quantile) earliest eclosing apple (17 males and 31 females) and hawthorn flies (10 males and 38 females) and 48 latest eclosing (≥ 97% quantile) apple (20 males and 28 females) and hawthorn flies (15 males and 33 females) were genetically scored by genotyping-by-sequencing (double digest restriction site associated DNA sequencing).

**Genome structure of *Rhagoletis pomonella***

*Rhagoletis pomonella* has a highly-structured genome that both complicates analysis of the results and may help enhance the adaptive tracking of the fly to varying environmental conditions by packaging together favorable combinations of eclosion time alleles in alternate chromosomal rearrangements (Feder et al. 2003a, b; Michel et al. 2010). DNA sequencing studies suggest that inversion polymorphism on at least three of these chromosomes originated in the Eje Volcánico Trans Mexicano region of central Mexico (EVTM) ~ 1.5 Mya and may be present in other fly populations due to subsequent secondary contact and gene flow (Feder et al. 2003a, 2005; Xie et al. 2007; Michel et al. 2007, 2010). The packaging of genes in inversions may have helped facilitate the rapid divergence and evolution of new races and species of *Rhagoletis* through the assembly of these blocks in novel combinations to colonize new hosts plants with differing fruiting times (Feder et al. 2005; Xie et al. 2008). In this regard, previous studies (Feder et al. 2003b; Egan et al. 2015; Ragland et al. 2017) have provided evidence for inversion polymorphism on all five of the major chromosomes constituting the *R. pomonella* genome (the fly also possesses a small, heterochromatic dot 6^th^ chromosome that presently has no markers). Results from Egan et al. (2015) and Ragland et al. (2017) imply that chromosomes 1, 3, 4, and 5 contain large inversions covering much of their lengths. Linked SNPs displaying high levels of linkage disequilibrium (LD) with one another (pairwise standardized composite disequilibrium values of r^2^ > 0.6; Weir 1979), were found distributed along the lengths of each of these four chromosomes, sometimes separated by map distances of up to several hundred centi-Morgans. Interspersed among the high LD SNPs were loci showing lower levels of LD both amongst themselves and with the high LD SNPs. These data may be explained by the high LD SNPs being associated with large alternate rearrangements on chromosomes 1, 3, 4, and 5, with gene flux (double recombination and gene conversion) shuffling lower LD SNPs between the inversions. Occasionally alleles segregating for high LD SNPs may be present in the alternate (“wrong”) arrangement, resulting in homokaryotypic test crosses involving such chromosomes indicating that high LD SNPs can sometimes be separated by substantial map distances. In contrast to chromosomes 1, 3, 4 and 5, eight different groups of high LD SNPs were resolved on chromosome 2. These eight high LD groups presumably represent different smaller-sized inversions on chromosome 2. Unfortunately, we have not yet been able to recombinationally map enough SNPs belonging to these eight groups to discern their physical distribution along chromosome 2.

As described in Ragland et al. (2017), LD will contribute to genetic correlations and will also affect adaptive divergence and restrict gene flow during ecological speciation, especially if structural features such as inversions inhibit recombination among selected blocks of genes (Kirkpatrick and Barton 2006; Lowry and Willis 2010; Joron et al. 2011; Feder et al. 2014; Flaxman et al. 2014). This appears to be the case in *R. pomonella*, where markers, including allozymes, cDNA, and microsatellites, that are associated with host divergence appear to often reside in regions inferred by genetic crosses to contain inversions (Feder et al. 2003; Michel et al. 2010). Thus, we examined subsets of SNPs mapping to the five major linkage groups of the *R. pomonella* genome divided into three different categories displaying high, intermediate, and low levels of composite LD (Weir 1979) with each other, as designated in Ragland et al. (2107), to investigate the effects of linkage and potential inversion polymorphism on diapause phenotypes and host-related divergence. The program LDna (linkage disequilibrium network analysis) developed by Kemppainen et al. (2015) was used to divide the mapped SNPs into the three different LD classes based on the criteria that all linked high LD SNPs displayed a r^2^ value of > 0.6 with at least one other member of the group, while no low LD SNP had a r^2^ value > 0.15 with any other SNPs residing on the same chromosome. Intermediate LD SNPs possessed r^2^ values ranging from 0.15 to 0.6 with linked loci. We note that the core conclusions of the study presented below do not rely on inversions, per se, just on LD. Thus, if future results find that some of the patterns we describe are due to particularly strong selection and low recombination occurring for certain co-linear regions of the genome rather than inversions, this would not undermine the arguments about the diapause traits.

**References Cited in Supplementary Information and Tables**

Egan, S. P., G. J. Ragland, L. Assour, T. H. Q. Powell, G. R. Hood, S. Emrich, P. Nosil, and J. L. Feder. 2015. Experimental evidence of genome-wide impact of ecological selection during early stages of speciation-with-gene-flow. Ecol. Lett. 18:817–25.

Feder, J. L., S. H. Berlocher, J. B. Roethele, H. Dambroski, J. J. Smith, W. L. Perry, V. Gavrilovic, K. E. Filchak, J. Rull, and M. Aluja. 2003a. Allopatric genetic origins for sympatric host-plant shifts and race formation in *Rhagoletis*. Proc. Natl. Acad. Sci. U. S. A. 100:10314–10319.

Feder, J. L., C. A. Chilcote, and G. L. Bush. 1988. Genetic Differentiation between Sympatric Host Races of *Rhagoletis pomonella*. Nature 336:61-64.

Feder, J. L., P. Nosil, and S. M. Flaxman. 2014. Assessing when chromosomal rearrangements affect the dynamics of speciation: implications from computer simulations. Front. Genet. 5:295.

Feder, J. L., J. B. Roethele, K. Filchak, J. Niedbalski, and J. Romero-Severson. 2003b. Evidence for inversion polymorphism related to sympatric host race formation in the apple maggot fly, Rhagoletis pomonella. Genetics 163:939–953.

Feder, J. L., X. Xie, J. Rull, S. Velez, A. Forbes, B. Leung, H. Dambroski, K. E. Filchak, and M. Aluja. 2005. Mayr, Dobzhansky, and Bush and the complexities of sympatric speciation in *Rhagoletis*. Proc. Natl. Acad. Sci. U. S. A. 102:6573–80.

Flaxman, S. M., A. C. Wacholder, J. L. Feder, and P. Nosil. 2014. Theoretical models of the influence of genomic architecture on the dynamics of speciation. Mol. Ecol. 23:4074–4088.

Hood, G. R., A. A. Forbes, T. H. Q. Powell, S. P. Egan, J. J. Smith, and J. L. Feder. 2015. [Sequential divergence and the multiplicative origin of community diversity](https://scholar.google.com/citations?view_op=view_citation&hl=en&user=5Vbw_V8AAAAJ&sortby=pubdate&imq=Jeffrey+L.+Feder&citation_for_view=5Vbw_V8AAAAJ:kRWSkSYxWN8C). Proceedings of the National Academy of Sciences U.S.A. doi:10.1073/pnas. 1424717112.

Joron, M., L. Frezal, R. T. Jones, N. L. Chamberlain, S. F. Lee, C. R. Haag, A. Whibley, M. Becuwe, S. W. Baxter, L. Ferguson, P. A. Wilkinson, C. Salazar, C. Davidson, R. Clark, M. A. Quail, H. Beasley, R. Glithero, C. Lloyd, S. Sims, M. C. Jones, J. Rogers, C. D. Jiggins, and R. H. Ffrench-Constant. 2011. Chromosomal rearrangements maintain a polymorphic supergene controlling butterfly mimicry. Nature 477:203–206.

Kemppainen, P., C. G. Knight, D. K. Sarma, T. Hlaing, A. Prakash, Y. N. Maung Maung, P. Somboon, J. Mahanta, and C. Walton. 2015. Linkage disequilibrium network analysis (LDna) gives a global view of chromosomal inversions, local adaptation and geographic structure. Mol. Ecol. Resour. 15:1031–1045.

Kirkpatrick, M., and N. Barton. 2006. Chromosome inversions, local adaptation and speciation. Genetics 173:419–34.

Lowry, D. B., and J. H. Willis. 2010. A widespread chromosomal inversion polymorphism contributes to a major life-history transition, local adaptation, and reproductive isolation. PLoS Biol. 8:e1000500.

Lyons-Sobaski, S., and S. H. Berlocher. 2009. Life history phenology differences between southern and northern populations of the apple maggot fly, *Rhagoletis pomonella*. Entomol. Exp. Appl. 130:149–159.

Michel, A.P., J. Rull, M. Aluja, and J. L. Feder. 2007. The Genetic Structure of Hawthorn-infesting *Rhagoletis pomonella* Populations in Mexico: Implications for Sympatric Host Race Formation. Molecular Ecology 16: 867-2878

Michel, A. P., S. Sim, T. H. Q. Powell, M. S. Taylor, P. Nosil, and J. L. Feder. 2010. Widespread genomic divergence during sympatric speciation. Proc. Natl. Acad. Sci. U. S. A. 107:9724–9729.

Ragland, G. J., M. M. Doellman, P. J. Meyers, G. R. Hood, S. P. Egan, T. H. Q. Powell, D. A. Hahn, P. Nosil, and J. L. Feder. 2017. A test of genomic modularity among life-history adaptations promoting speciation with gene flow. Mol. Ecol. 26:3926–3942.

Weir, B. S. 1979. Inferences about linkage disequilibrium. Biometrics 35:235–254.

Xie, X., A. P. Michel, D. Schwarz, J. Rull, S. Velez, A. A. Forbes, M. Aluja, and J. L. Feder. 2008. Radiation and divergence in the *Rhagoletis pomonella* species complex: inferences from DNA sequence data. J. Evol. Biol. 21:900–913.

Xie, X., J. Rull, A. P. Michel, S. Velez, A. A. Forbes, N. F. Lobo, M. Aluja, and J. L. Feder. 2007. Hawthorn-infesting populations of *Rhagoletis pomonella* in Mexico and speciation mode plurality. Evolution 61:1091–1105.
